# Announcement

**Published:** 1891-03

**Authors:** 


### ANNOUNCEMENT.

With this issue of the JOURNAL Dr. Luther B. Grandy, who has purchased an interest, will become actively connected with its editorial management. Dr. Grandy is one of the brainiest and most competent young men in the profession. We feel sure the accession of Dr. Grandy will prove of great benefit to the JOURNAL.

